# Traumatic Arthrotomy with Pneumarthrosis on Plain Radiograph of the Knee

**DOI:** 10.5811/westjem.2015.12.29317

**Published:** 2016-03-02

**Authors:** Timothy D. Roberts

**Affiliations:** Starship Children’s Hospital, Department of Paediatric Orthopaedics, Auckland, New Zealand

An eight-year-old boy presented to the emergency department (ED) with a 2cm-long laceration over the prepatellar region of his left knee after falling over and cutting his knee on broken glass. Physical examination demonstrated the laceration breached the dermis but otherwise there was no obvious defect in the deep fascial layer. He had a free range of motion of his knee and clinically his extensor mechanism was intact; however, a plain lateral radiograph showed that he had pneumarthrosis of his knee joint. Within six hours of injury the wound was formally explored in the operating room and a small breach in the knee capsule was found. The wound edges were debrided, the knee joint irrigated and the skin closed primarily. Following surgery he received 24 hours of antibiotic coverage with a first-generation cephalosporin.

Gas in the joint or “pneumarthrosis” in the context of trauma is not uncommon around superficial joints such as the knee and indicates that there is a penetrating wound with intra-articular extension.^1^ This implies that the joint has been inoculated with bacteria and pyoarthrosis is possible if not managed appropriately.^1^ The standard treatment for a traumatic arthrotomy is antibiotic coverage and prompt surgical debridement in the operating room with lavage of the soft tissues and joint.^2^ In the absence of gross contamination primary closure can be performed.^2^

Even innocuous-looking lacerations adjacent to or overlying superficial joints can lead to a traumatic arthrotomy. Plain radiographs should be obtained and a high suspicion for intra-articular extension maintained. For lacerations around superficial joints there should be a low threshold for exploring and washing these wounds out in the operating room rather than closing primarily in the ED.

## Figures and Tables

**Figure:**
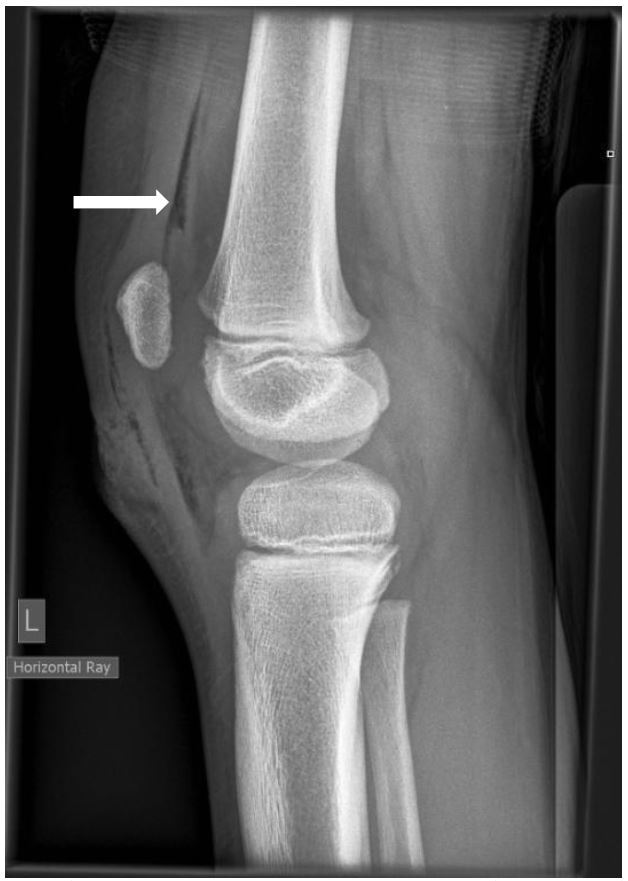
Lateral radiograph of knee showing gas in the suprapatellar pouch of the joint capsule.
